# Supplementary material for: HLH-11 modulates lipid metabolism in response to nutrient availability
Source: Nat Commun. 2020 Nov 24;11:5959. doi: 10.1038/s41467-020-19754-1 (PMC7686365; doi:10.1038/s41467-020-19754-1)
Supplement: Supplementary file 1 — Supplementary information [file 41467_2020_19754_MOESM1_ESM.pdf]

Supplementary Information

HLH-11 modulates lipid metabolism in response to nutrient availability

Li Y. et al.

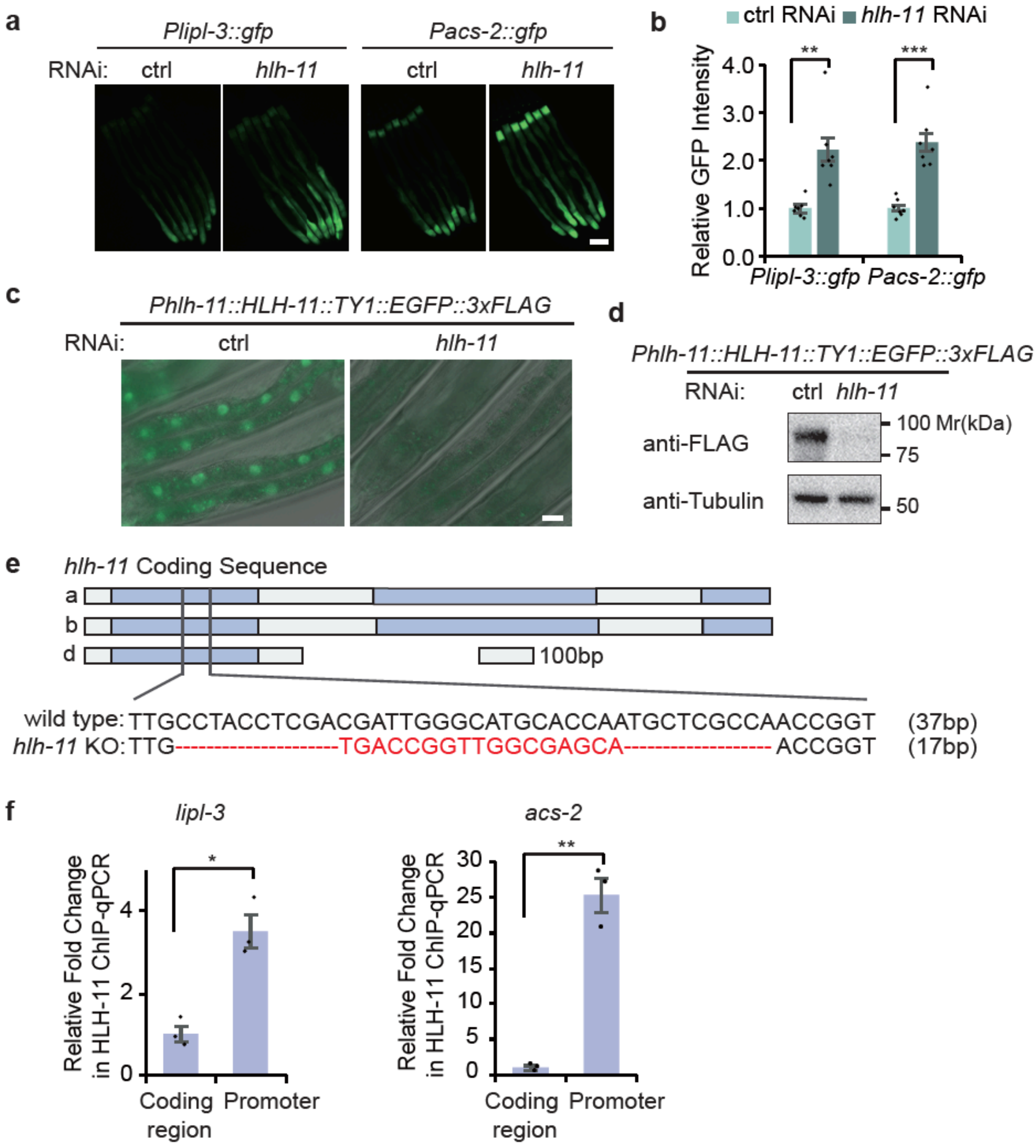

Supplementary Figure 1. Knockdown of *hlh-11* activates transcription of *lipl-3* and *acs-2*. **a** Representative fluorescence images of *Plipl-3::gfp* and *Pacs-2::gfp* worms fed with control RNAi or *hlh-11* RNAi. Scale bar, 100 µm. **b** Quantification of GFP intensity in (a). n=6 worms examined per condition. **p=0.0049; ***p=0.0004. **c** Representative fluorescence images of *Phlh-11::HLH-11::TY1::EGFP::3xFLAG* worms fed with control or *hlh-11* RNAi. Scale bar, 20 µm. Three independent experiments were performed with similar results. **d** Immunoblotting images showing protein levels of HLH-11 and Tubulin (loading control) in *Phlh-11::HLH-11::TY1::EGFP::3xFLAG* transgenic worms fed with control or *hlh-11* RNAi. Two independent experiments were performed with similar results. **e** Diagram depicting the *hlh-11* knockout strain generated via the CRISPR/Cas9 approach. **f** ChIP-qPCR analysis of young adult worms expressing *Phlh-11::HLH-11::TY1::EGFP::3xFLAG*. Anti-GFP antibody was utilized for immunoprecipitation. qPCR was conducted using primers targeting the coding region or promoter region of *acs-2* or *lipl-3*. n=3 biologically independent samples. *p=0.013; **p=0.009. Error bars indicate mean ± SEM. Statistical analyses were performed by Student's t-test (unpaired, two-tailed), *p< 0.05; **p< 0.01. Source data are provided as a Source Data file.

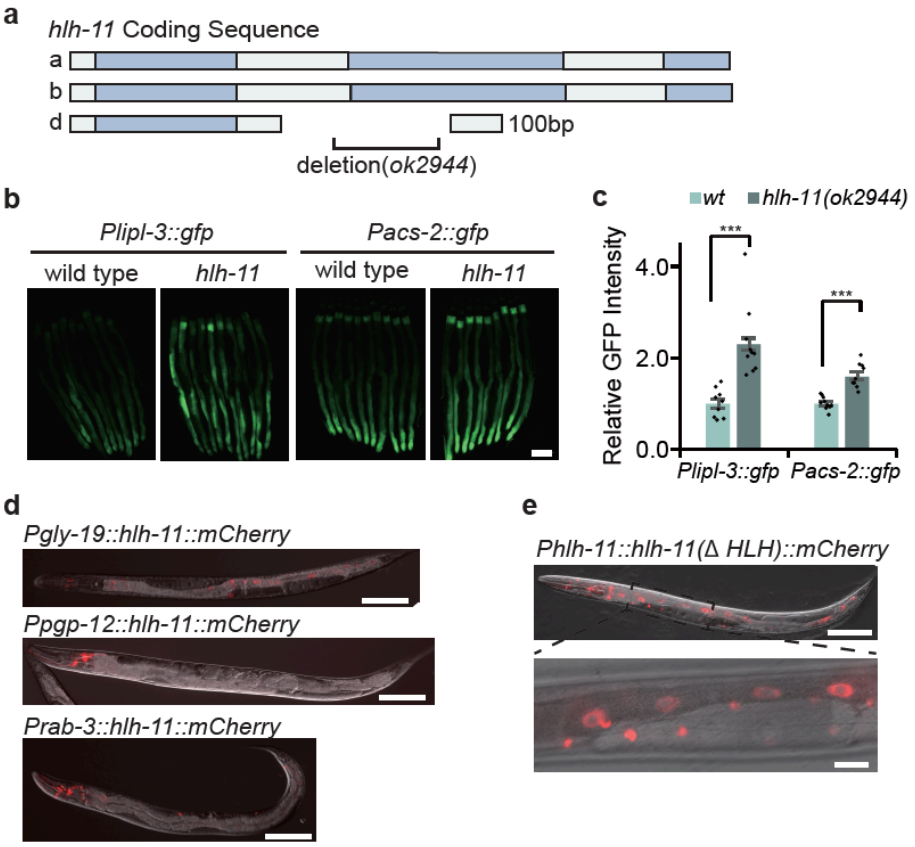

Supplementary Figure 2. *hlh-11* knockouts activate transcription of *lipl-3* and *acs-2*. a Diagram depicting the *hlh-11*(ok2944) deletion. **b** Representative fluorescence images of *Plipl-3::gfp* and *Pacs-2::gfp* reporters in wild-type worms or *hlh-11(ok2944)* mutants. Three independent experiments were performed with similar results. Scale bar, 100 µm. **c** Quantification of GFP intensity in (c). n=9-10 worms examined per condition. ***p=0.0004 (left), ***p=6.5E-5 (right). **d** Representative fluorescence images of HLH-11::mCherry driven by the intestine-specific promoter *gly-19*, the excretory cell promoter *pgp-12*, or the pan-neuronal promoter *rab-3*. n=6 worms were examined with similar results. Scale bar, 200 µm. **e** Expression pattern of HLH-11(Δ HLH)::mCherry. n=6 worms were examined with similar results. Scale bar, 200 µm (upper), 20 µm (lower). Error bars indicate mean ± SEM. Statistical analyses were performed by Student's t-test (unpaired, two-tailed), *p< 0.05; **p< 0.01. Source data are provided as a Source Data file.

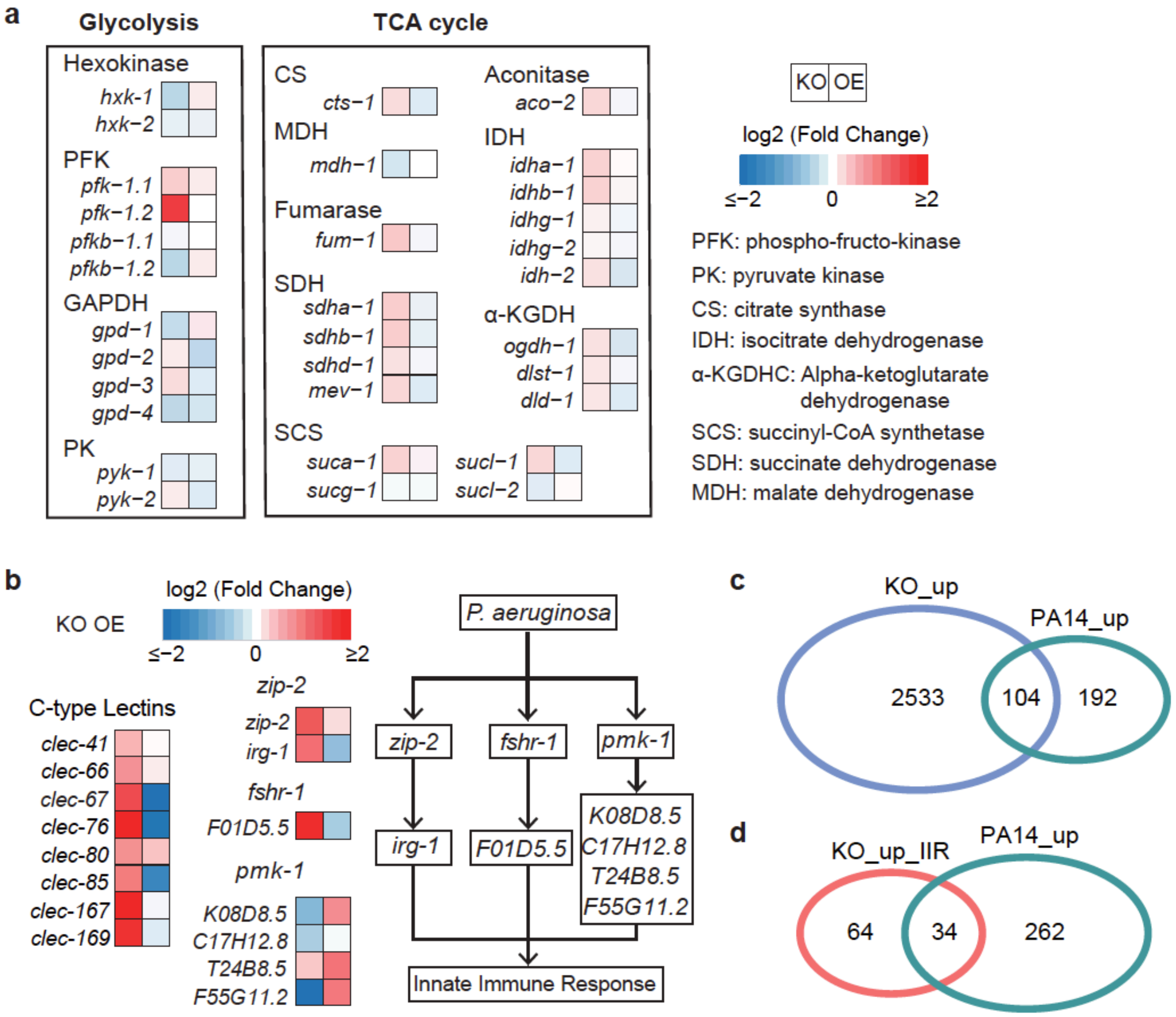

Supplementary Figure 3. Knockout of *hlh-11* activates the innate immune response. a Heat map of fold changes in transcript levels of glycolysis and TCA cycle genes in *hlh-11* KO or OE worms. The fold changes were calculated by dividing the transcript level of each indicated gene in *hlh-11* KO or OE by that of wild-type. **b** Heat map of fold changes in transcript levels of immune genes in *hlh-11* KO or OE worms. The fold changes were calculated by dividing the transcript level of each indicated gene in *hlh-11* KO or OE by that of wild-type. **c** Venn diagram showing the overlap of up-regulated genes in *hlh-11* KO and in wild-type animals upon PA14 infection. **d** Venn diagram showing the overlap of up-regulated innate immunity response (IIR) genes in *hlh-11* KO and in wild-type animals upon PA14 infection. Source data are provided as a Source Data file.

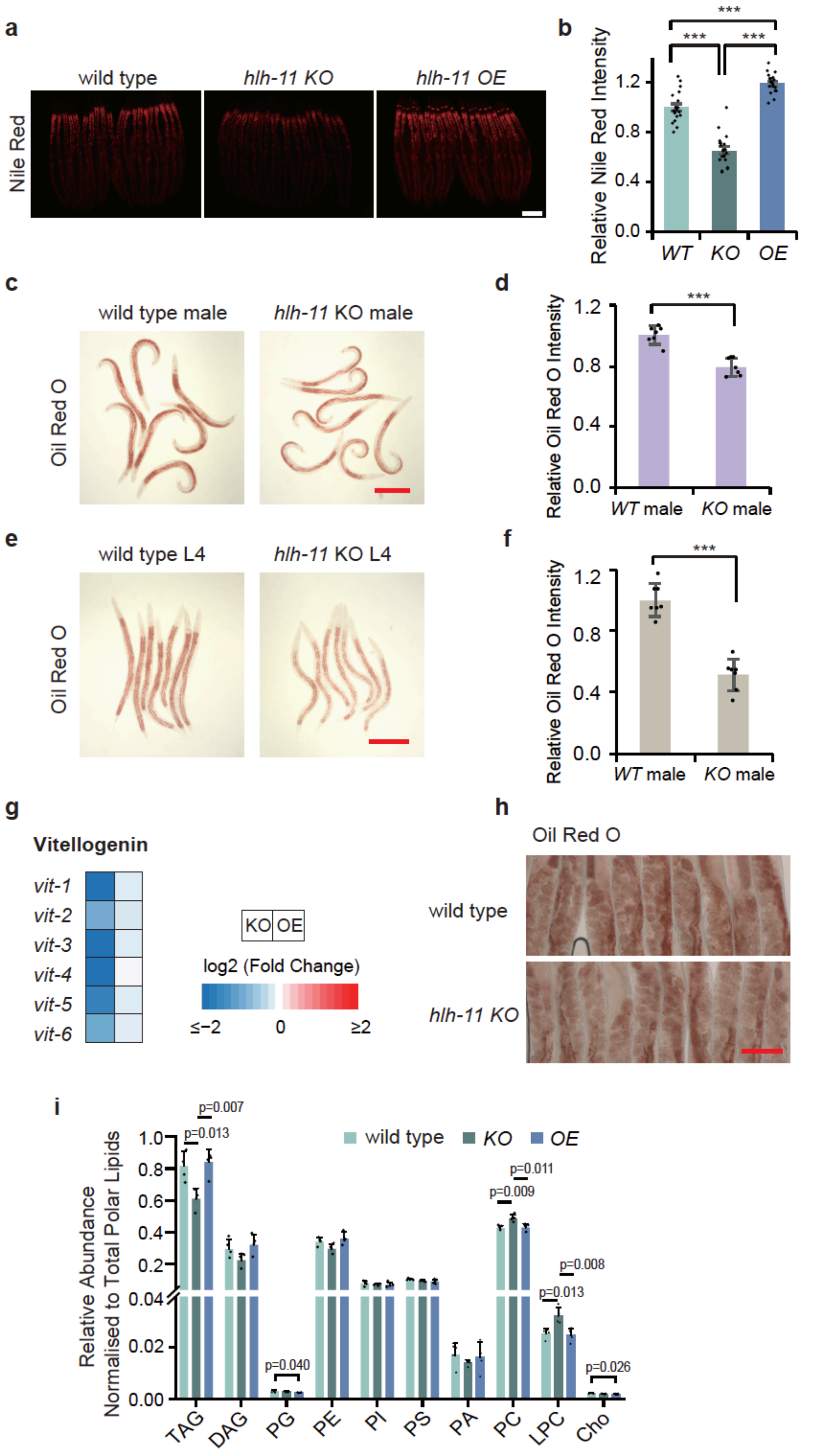

Supplementary Figure 4. Knockout of *hlh-11* affects lipid abundance. a Nile Red staining of wild-type, *hlh-11* KO and *hlh-11* OE worms. Scale bar, 200µm. **b** Quantification of Nile Red signals in (a). Error bars indicate mean ± SEM. Statistical analyses were performed by Student's t-test (unpaired, two-tailed). n=20 worms were examined per condition. ***p=7.2E-11 (KO vs WT), ***p=9.0E-7 (OE vs WT), ***p=3.0E-18 (OE vs KO). **c, e** Oil Red O staining of male (c) or L4 (e) wild-type and *hlh-11* KO worms. Scale bar, 200 µm. **d, f** Quantification of Oil

Red O signals from upper intestine in (c, e). Error bars indicate mean ± SEM. Statistical analyses were performed by Student's t-test (unpaired, two-tailed). n=7 worms were examined per condition. ***p=2.5E-5 (d); ***p=1.8E-6 (f). **g** Heat map of fold changes in transcript levels of vitellogenin genes in *hlh-11* KO or OE worms. The fold changes were calculated by dividing the transcript level of each indicated gene in *hlh-11* KO or OE by that of wild-type. **h** Representative images of Oil Red O staining revealing the eggs of wild-type and *hlh-11* KO worms (day-1 adult). Three independent experiments were performed with similar results. Scale bar, 100 µm. **i** Relative abundance of total TAG (triacylglycerols), DAG (diacylglycerols), PG (phosphatidylglycerols), PE (phosphatidylethanolamines), PI (phosphatidylinositols), PS (phosphatidylserines), PA (phosphatidic acids), PC (phosphatidylcholines), LPC (lyso-phosphatidylcholine), and Cho (free cholesterols) in wild-type, *hlh-11* KO and *hlh-11* OE worms. Error bars indicate mean ± SEM. Statistical analyses were performed using one-sided Tukey's HSD test. n=4 biologically independent samples. p values are shown in panel (i). Source data are provided as a Source Data file.

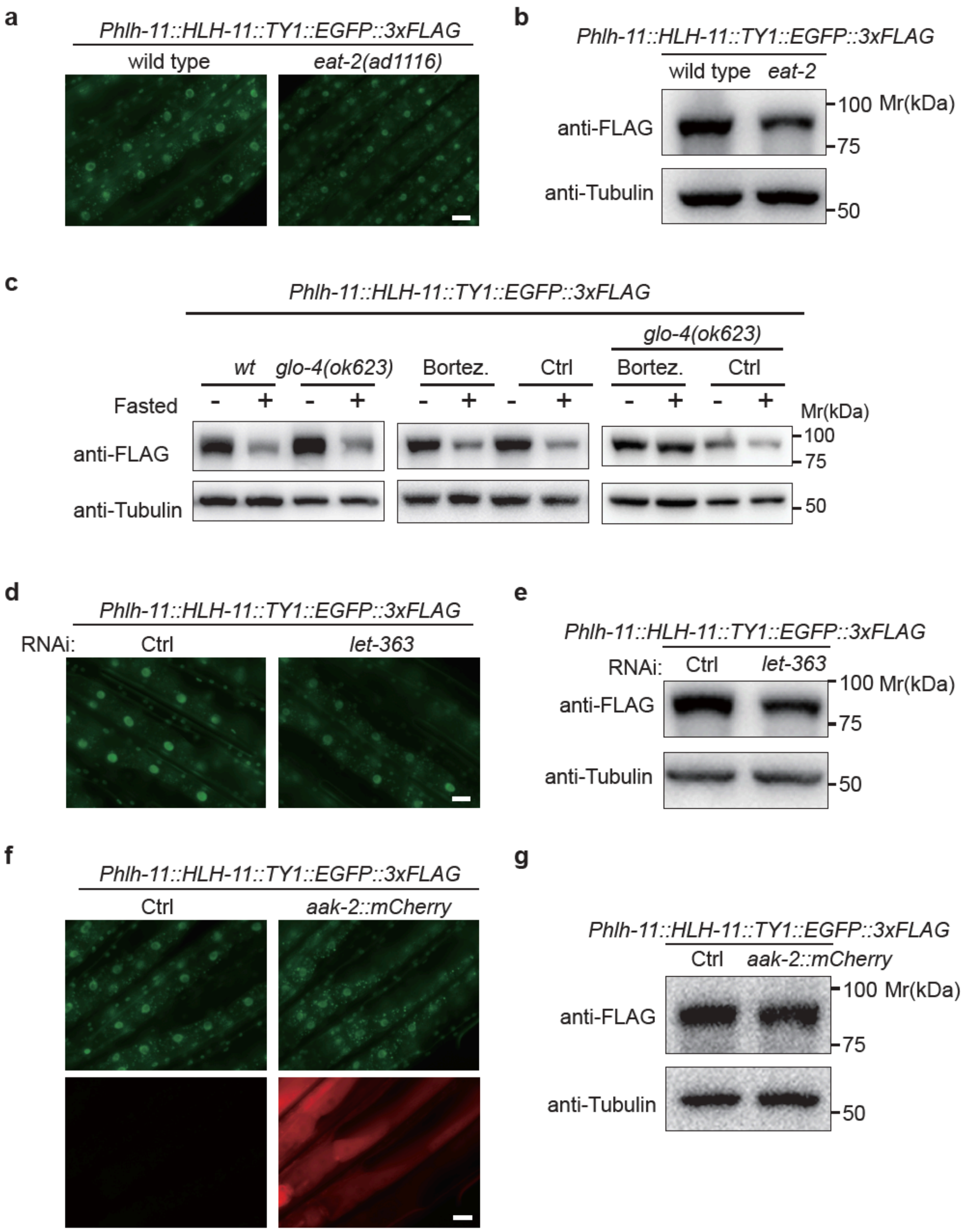

Supplementary Figure 5. Caloric restriction or mTOR inhibition activates the degradation of HLH-11. a Representative fluorescence images of the *Phlh-11::HLH-11::TY1::EGFP::3xFLAG* reporter in wild-type worms or *eat-2(ad1116)* mutants. Three independent experiments were performed with similar results. Scale bar, 20 µm. **b** Immunoblotting images showing protein levels of HLH-11 and Tubulin (loading control) in wild-type worms or *eat-2(ad1116)* mutants expressing *Phlh-11::HLH-11::TY1::EGFP::3xFLAG*. Two independent experiments were performed with similar results. **c** Immunoblotting images showing protein levels of HLH-11 and Tubulin (loading control) in worms with the indicated genotype under feeding or fasting conditions, and treated with or without the proteasome inhibitor Bortezomib (5 µg/ml). Three independent experiments were performed with similar results. **d** Representative fluorescence images of *Phlh-11::HLH-11::TY1::EGFP::3xFLAG* worms fed with control or *let-363* RNAi. Three independent experiments were performed with similar results. Scale bar, 20 µm. **e** Immunoblotting images showing protein levels of HLH-11

and Tubulin (loading control) in *Phlh-11::HLH-11::TY1::EGFP::3xFLAG* transgenic worms fed with control or *let-363* RNAi. Two independent experiments were performed with similar results. **f** Representative fluorescence images of the *Phlh-11::HLH-11::TY1::EGFP::3xFLAG* reporter in wild-type worms or AAK-2::mCherry overexpression worms. Three independent experiments were performed with similar results. Scale bar, 20 µm. **g** Immunoblotting images showing protein levels of HLH-11 and Tubulin (loading control) in wild-type or AAK-2::mCherry worms expressing *Phlh-11::HLH-11::TY1::EGFP::3xFLAG*. Two independent experiments were performed with similar results. Source data are provided as a Source Data file.

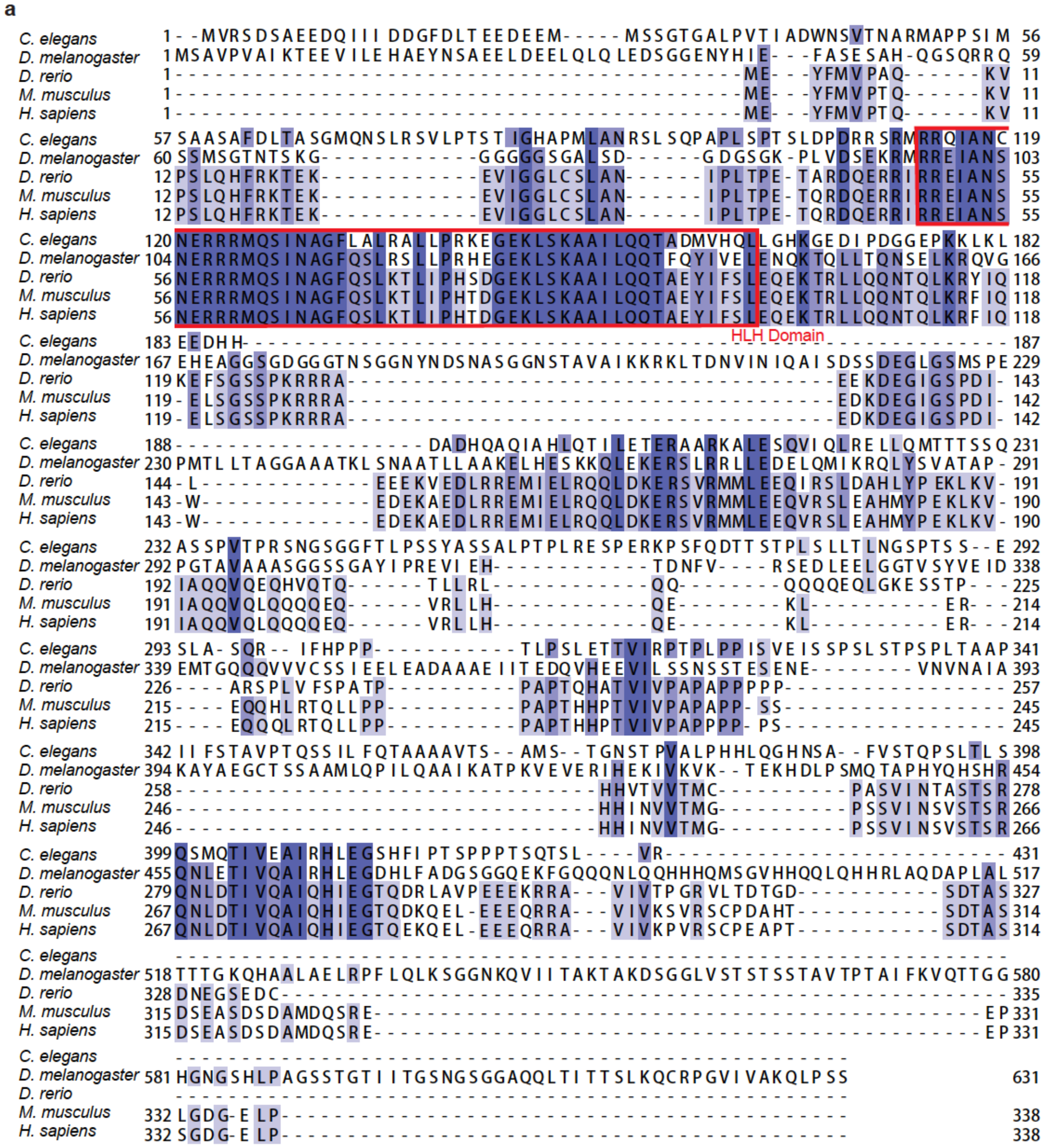

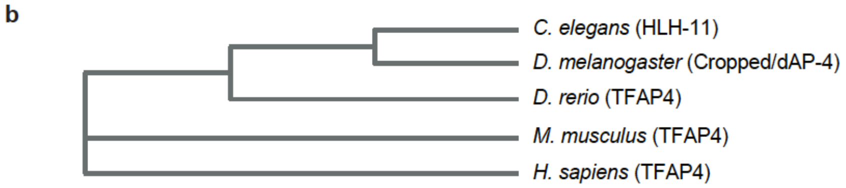

Supplementary Figure 6. HLH-11 is conserved across species. a Protein sequence alignment of HLH-11 and its orthologs in fruit fly (*D. melanogaster*), zebrafish (*D. rerio*), mouse (*M. musculus*) and human (*H. sapiens*). The HLH domain is highlighted with a red box. **b** Cladogram of HLH-11 family proteins in (a).

Supplementary Table 1: Primer Sequences used for RT-qPCR

	**Primer Sequence (5' – 3')**
ama-1 (C. elegans)	GACATTTGGCACTGCTTTGT
	ACGATTGATTCCATGTCTCG
acs-2 (C. elegans)	AAGGAGATGAGAATGACTGAT
	GTTCCGACATGGTGACTA
lipl-3 (C. elegans)	GTGCACTACTGCTCGTGATTC
	CATAGTCCAGTCTGTGGATGC
hlh-11 (C. elegans)	AGCACCCACTCATCATCCAC
	TTGCATCCATGTCGGGTGAG
actb (HepG2)	CAACGGTGAGCATATTGCCG
	CGCTCTTGTAGCGGATGAGA
tfap4 (HepG2)	TCGATTCACCACAGAACGCA
	AGCCATTTGCCAACCGTAGA
