# Supplementary material for: HLH-11 modulates lipid metabolism in response to nutrient availability
Source: Nat Commun. 2020 Nov 24;11:5959. doi: 10.1038/s41467-020-19754-1 (PMC7686365; doi:10.1038/s41467-020-19754-1)
Supplement: Supplementary file 3 — Description of Additional Supplementary Files [file 41467_2020_19754_MOESM3_ESM.docx]

Description of Additional Supplementary Files

File Name: Supplementary Data 1

Description: Lists of RNAi sub-libraries used in the screen

File Name: Supplementary Data 2

Description: List of 57 primary hits after RNAi Screening

File Name: Supplementary Data 3

Description: Lists of genes with differential expression in *hlh-11* KO or OE worms
