# Supplementary material for: HLH-11 modulates lipid metabolism in response to nutrient availability
Source: Nat Commun. 2020 Nov 24;11:5959. doi: 10.1038/s41467-020-19754-1 (PMC7686365; doi:10.1038/s41467-020-19754-1)

Protein gels or membranes were cutted into slices before incubation with primary antibodies.

The Precision Plus ProteinTM Dual Color Standards (Cat#161-0374) from BioRad was used as the protein marker.

Figure 5c

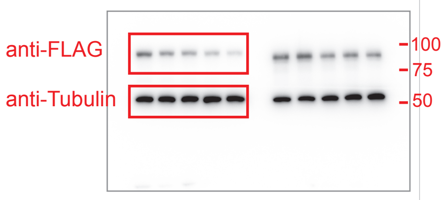

Figure 5d

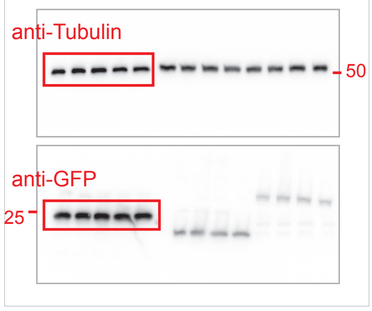

Figure 5g

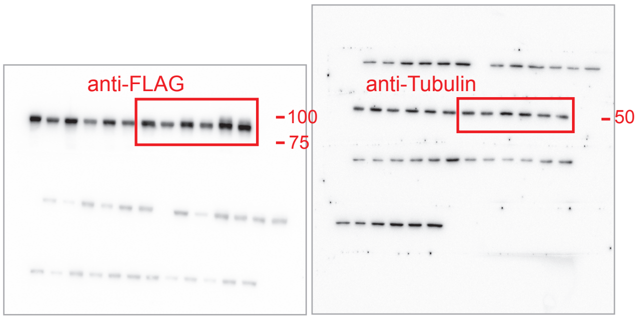

Figure 6d

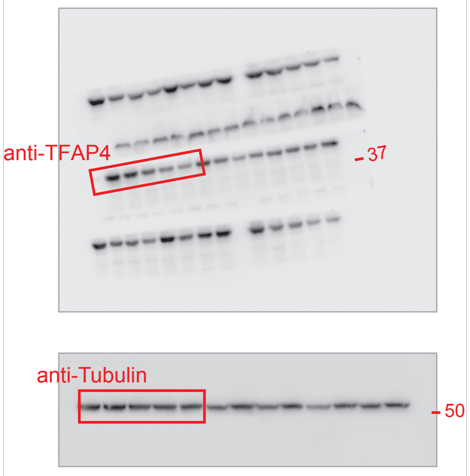

Figure 6f

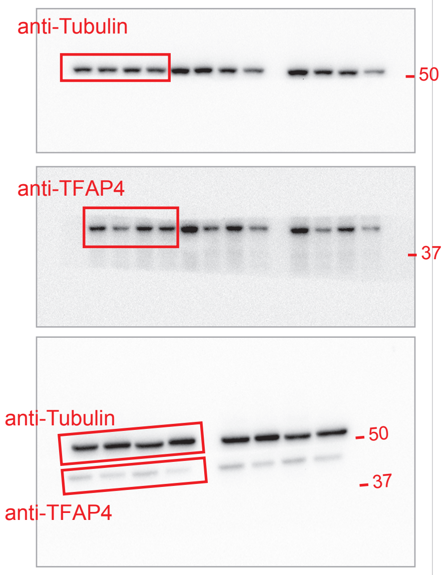

Figure 6g

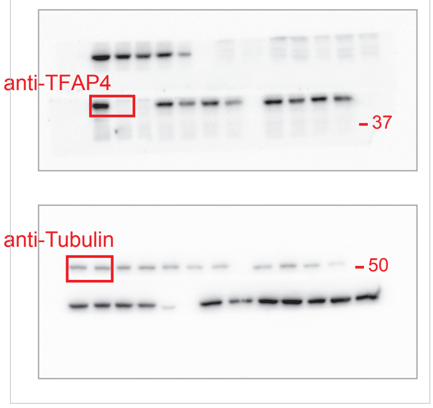

Supplementary Figure 1d

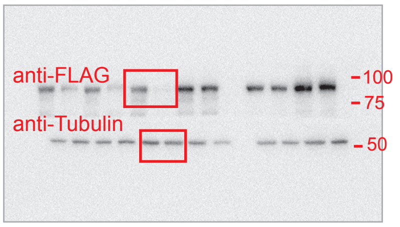

Supplementary Figure 5b

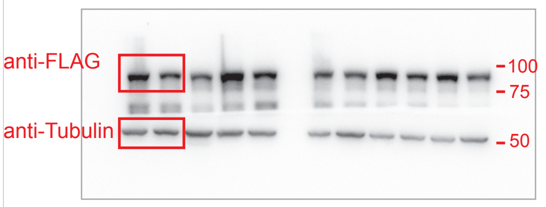

Supplementary Figure 5e

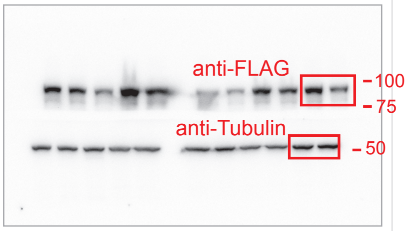

Supplementary Figure 5g

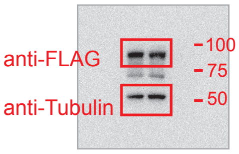

Supplementary Figure 5c

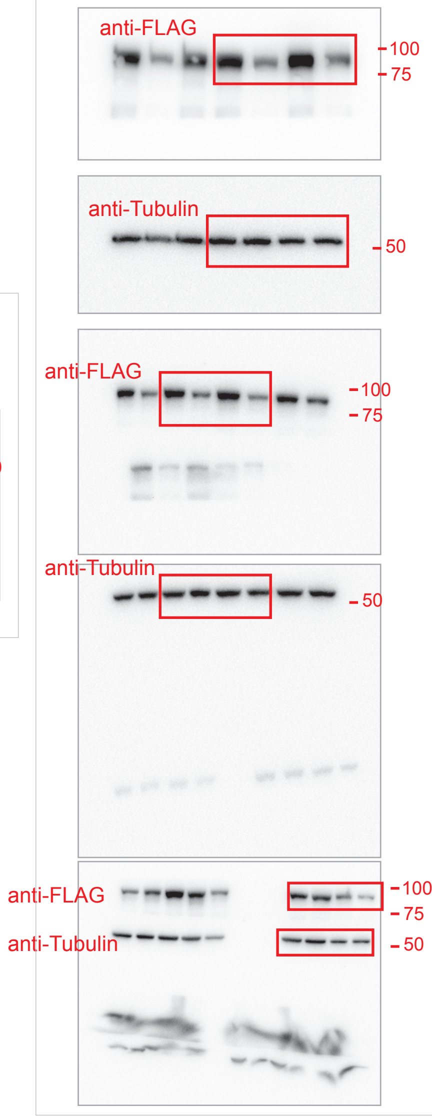

Supplement: Supplementary file 8 — Source Data [file 41467_2020_19754_MOESM8_ESM.zip › Source Data/Source Data-western blots.pdf]
